# Erythroid anion transport, nitric oxide, and blood pressure

**DOI:** 10.3389/fphys.2024.1363987

**Published:** 2024-04-10

**Authors:** Kate Hsu

**Affiliations:** ^1^ The Laboratory of Immunogenetics, Department of Medical Research, MacKay Memorial Hospital, New Taipei City, Taiwan; ^2^ MacKay Junior College of Medicine, Nursing, and Management, New Taipei City, Taiwan; ^3^ Institute of Biomedical Sciences, MacKay Medical College, New Taipei City, Taiwan

**Keywords:** band 3 (anion exchanger-1, AE1), GP.Mur (Miltenberger subtype III, Mi.III), nitric oxide, blood pressure, vasodilation, dipyridamole, glycophorin, erythrocyte

## Abstract

Glycophorin A and glycophorin B are structural membrane glycoproteins bound in the band 3 multiprotein complexes on human red blood cells (RBCs). Band 3 is an erythroid-specific anion exchanger (AE1). AE1-mediated HCO_3_^−^ transport provides the substrate for the enzyme-catalyzed conversion HCO_3_^−^_(aq)_ ⇌ CO_2(g)_, which takes place inside the RBCs. Bicarbonate transport via AE1 supports intravascular acid–base homeostasis and respiratory excretion of CO_2_. In the past decade, we conducted several comparative physiology studies on Taiwanese people having the glycophorin variant GPMur RBC type (which accompanies greater AE1 expression). We found that increased anion transport across the erythrocyte membrane not only enhances gas exchange and lung functions but also elevates blood pressure (BP) and reduces nitric oxide (NO)-dependent vasodilation and exhaled NO fraction (FeNO) in healthy individuals with GP.Mur. Notably, in people carrying the GPMur blood type, the BP and NO-dependent, flow-mediated vasodilation (FMD) are both more strongly correlated with individual hemoglobin (Hb) levels. As blood NO and nitrite (NO_2_^−^) are predominantly scavenged by intraerythrocytic Hb, and NO_2_^−^ primarily enters RBCs via AE1, could a more monoanion-permeable RBC membrane (i.e., GPMur/increased AE1) enhance NO_2_^−^/NO_3_^−^ permeability and Hb scavenging of NO_2_^−^ and NO to affect blood pressure? In this perspective, a working model is proposed for the potential role of AE1 in intravascular NO availability, blood pressure, and clinical relevance.

## Introduction

Hemoglobin (Hb) is the main scavenger of NO species in the mammalian vasculature (Lancaster, 1994; Patel et al., 2011). Band 3, an anion transporter with ∼ a million protein copies per human RBC, has been postulated to play a critical role in erythrocyte processing of NO species since late 1990s (Vaughn et al., 2000; Huang et al., 2001; Pawloski et al., 2001; Han et al., 2003; Kim-Shapiro et al., 2006; Jana et al., 2018; Premont et al., 2020). We came across this perplexing topic of research when we were conducting human exercise tests that examined the impacts of GP.Mur on respiration and unexpectedly found a significant association between GP.Mur and higher blood pressure. GP.Mur (a glycophorin B-A-B variant exclusively expressed on human RBCs) is a blood type unique to Southeast Asian (SEA) populations, with ∼4.7% prevalence in Taiwan and 2–23% in many areas of SEA, and is rare among non-SEA ethnic populations (e.g., Japanese and other ethnic groups of the Northeastern Asian origins) (Prathiba et al., 2002; Hsu et al., 2013; Wei et al., 2016; Jongruamklang et al., 2020; Yang et al., 2021; Kaito et al., 2022). The main molecular function of GP.Mur is to enhance AE1 protein expression on the RBC membrane (Hsu et al., 2009). Since the cytoplasmic domain of AE1 binds hemoglobin (preferentially deoxyHb) in the submembranous interface and deoxyHb also functions as a nitrite reductase that produces nitric oxide (Walder et al., 1984; Gladwin et al., 2009), we hypothesized that AE1 might be involved in regulation of blood pressure through intraerythrocytic Hb–NOx reactions.

## Blood pressure and NO-dependent vasodilation are affected by GP.Mur/increased AE1

From the respiratory/exercise physiology studies conducted on healthy adults and professional athletes, we repeatedly observed a slightly but statistically significant higher systolic blood pressure (SBP) among those carrying the GP.Mur blood type (Hsu et al., 2015; Chen et al., 2022; Hsu et al., 2023). For verification, we collaborated with one of our health check-up centers located in Eastern Taiwan, where the GP.Mur+ population is four times higher than that in other regions of Taiwan (Chen et al., 2022). We selected 989 Eastern Taiwanese residents under 55 years of age (one-fifth of them were GP.Mur+) whose clinical laboratory data from the annual health check-up were within the normal ranges and who were not on any drug treatments, including antihypertensives. Among them, the non-diseased men and women with GP.Mur indeed presented 3–5 mmHg higher systolic blood pressure (SBP) than the negative control people (Chen et al., 2022). Intriguingly, in this study, the incidence of early-onset hypertension (BP > 130/80 mmHg in those ≤45 years old) is significantly higher among GP.Mur+ carriers. Young men and women under 45 years of age are 1.6-fold and 3.4-fold more likely to present hypertension than age-matched controls living in the same area. As early-onset hypertension is largely hereditary (Suvila et al., 2020), this type of early-onset hypertension found among GP.Mur+ carriers is unlikely to be mainly due to unhealthy lifestyles (“GP.Mur-unique hypertension”).

To test our hypothesis that AE1-Hb could contribute to “GP.Mur-unique hypertension” via AE1–Hb–NO reactions in RBCs, we examined the impacts of GP.Mur/more AE1 on NO-dependent vasodilation in male college athletes. We measured their flow-mediated vasodilation (FMD, reflecting NO-mediated vasodilation (Green et al., 2014)) and nitroglycerin-induced, NO-independent vasodilation (NID) in this homogeneous population. Ultrasound was used to track vasodilation or the degree of increase in the brachial arterial diameter of each subject during a period of stimulation and recovery (FMD was stimulated by 5-minute vasoconstriction and NID by sublingual nitroglycerin). While NID in GP.Mur+ and the control athletes was not different, FMD measured in the GP.Mur+ athletes was significantly lower than FMD measured in the control athletes (4.6% ± 2.3% [GP.Mur] versus 6.3% ± 2.4% [control]) (Hsu et al., 2021).

## Stronger dependence of FMD and BP on Hb levels in GP.Mur+ people

**FIGURE 1:**
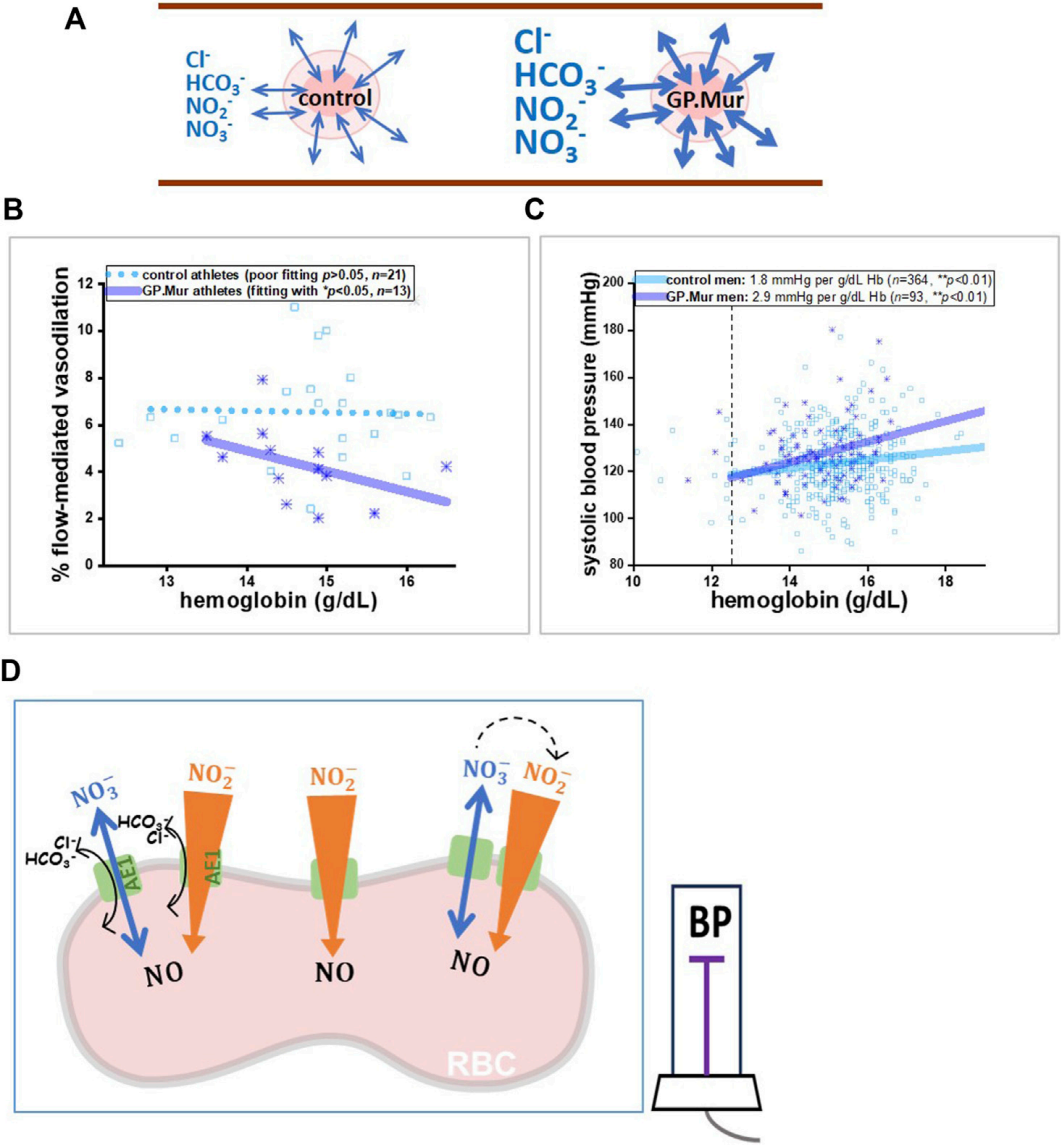
A model illustrating that GP.Mur/higher AE1 expression could increase NOx metabolic cycling and NO scavenging by intraerythrocytic Hb. **(A)** The bidirectional arrows indicate anion fluxes (mostly HCO_3_^−^, Cl^−^, NO_2_^−^, and NO_3_^−^) via AE1 (Jennings, 2021). The GP.Mur+ RBC membrane is more permeable to these monoanions (shown as thicker bidirectional arrows). **(B, C)** Previous studies on healthy people showed that GPMur/increased AE1 imposes higher sensitivities of FMD and SBP to Hb levels. **(B)** The Hb–FMD correlation for GP.Mur+ male athletes (*R* ∼ 0.53) was replotted using the published data (Hsu et al., 2021). **(C)** The Hb–SBP correlations for GP.Mur+ men (*R* ∼ 0.44) and for control men (*R* ∼ 0.37) were replotted using the published data collected at the MMH health check-up center in Eastern Taiwan (Chen et al., 2022). These GP.Mur comparative human studies revealed that GP.Mur/higher AE1 expression reduces NO-dependent FMD and increases blood pressure. **(D)** In the proposed model, AE1 (green gate) transports nitrite (orange-colored arrow) and nitrate (blue arrow) across the red cell membrane. Nitrite influx is followed by rapid and complex redox reactions catalyzed by Hb inside the RBCs, and thus the large concentration gradient of nitrite across the RBC membrane should favor nitrite influx (Chen et al., 2024). Nitrate is generally synthesized from excessive NO by oxyHb and is a stable reservoir of NO that permeates in or out of the RBCs via AE1 following its concentration gradient (bidirectional flux of nitrate). If depletion of plasma nitrite could accelerate the enzymatic conversion of extracellular nitrate to nitrite by nitrate reductase and xanthine oxidoreductase (dotted arrows), this may help explain how GP.Mur/higher AE1 lowers systemic NO and triggers high BP.

From a seminal study on Andean high-altitude dwellers with excessive erythrocytosis (EE), a significantly inverse correlation was found between Hb and NO-dependent vasodilation (%FMD) in EE patients (Tremblay et al., 2019). In these GP.Mur+ athletes, we also observed a significantly inverse correlation between Hb and %FMD (Figure 1B: the correlation is shown as the solid line); this correlation was not found in the control athletes (Figure 1B: shown as a dotted line as the correlation was not statistically significant) (Hsu et al., 2021). A closer comparison between Tremblay’s and our data revealed a key difference: Hb in the highlanders with EE was abnormally high (20–27 g/dL), and that for the male college athletes in this study with or without GP.Mur was all within the normal range for men (12.5–17 g/dL). The inverse correlation of Hb-FMD was not found in our GPMur-negative control athletes or in the Andean non-patient highlander subjects whose Hb levels were below 21 g/dL (Tremblay et al., 2019). Hb is a major NO scavenger, and abnormally high Hb concentrations (accompanied with an abnormally higher RBC count) undoubtedly scavenge more NO in the vasculature and result in lower FMD (as in the EE cases). In contrast, the significant Hb–FMD inverse correlation found within the normal range of Hb in the GP.Mur+ subjects indicates that their NO-dependent vasodilation is more sensitive to intravascular Hb concentrations (Figure 1B).

Hb and blood pressure are weakly but directly correlated. The direct correlation of Hb and BP was first identified in half a million Dutch blood donors at Sanquin: an increase of 1 g/dL Hb accompanies an increase of ∼0.81 mmHg SBP among Dutch men (Atsma et al., 2012). The degrees of the Hb–BP correlation vary slightly between men and women or among different ethnic populations (Gobel et al., 1991; Kawamoto et al., 2012; Lee et al., 2015; Xuan et al., 2019; Abumohsen et al., 2021).

In our population study conducted at the health check-up center in Eastern Taiwan, an increase of 1 g/dL Hb accompanies a ∼2.9 mmHg increase of SBP in GP.Mur+ men, compared to an ∼1.8 mmHg increase of SBP in the control (GP.Mur-negative) men, after controlled for age and BMI by multivariate regression (Figure 1C) (Chen et al., 2022). Compared to the controls, the blood pressure in the GP.Mur+ men was ∼60% more dependent on individual Hb levels. The positive correlation between Hb and BP could be caused by the following: (1) more circulating RBCs (as shown with an abnormally higher RBC count and Hb concentrations in high-altitude EE) increase blood viscosity and intravascular pressure (Tremblay et al., 2019), and (2) Hb scavenges intravascular NO species more effectively (as shown with a stronger Hb–FMD correlation and stronger Hb–BP correlation found in GP.Mur+ carriers) (Figure 1B, C) (Hsu et al., 2021; Chen et al., 2022). Since Hb is enclosed inside the RBC membrane, the second scenario points to the fact that GP.Mur/increased AE1 activities on the RBC membrane could promote Hb scavenging of NO species during blood circulation.

## Toward developing a hypothesis that increased AE1 expression could reduce systemic NO

A higher monoanion permeability of the GP.Mur+ RBC membrane enhances the NO_2_^−^/NO_3_^−^ flux across the RBC membrane and intraerythrocytic processing of NO species (Figure 1A, D). NO_2_^−^ and NO_3_^−^ (nitrate) are the major constituents of the intravascular NO reservoir. Excessive NO in the vasculature is mainly converted to nitrate by oxygenated Hb (NO + oxyHb → NO_3_^−^ + metHb). Compared to nitrite, nitrate is a more stable and much less reactive NO metabolite. Both NO_2_^−^ and NO_3_^−^ freely permeate across the RBC membrane through AE1 (Jennings, 2021; Chen et al., 2024). The more reactive nitrite (half-life ∼0.5 h compared to 5–8 h of nitrate) tends to be converted to NO in a less oxygenated environment, e.g., arterioles. This reaction stimulated by hypoxia (NO_2_^−^ + deoxyHb → NO + metHb) facilitates vasodilation and blood flow (Cosby et al., 2003; Dejam et al., 2005). In the case of GP.Mur, enhanced nitrite flux helps facilitate deoxyHb-catalyzed reduction of nitrite to NO gas. NO, with a half-life of ∼2 msec, dissipates extremely rapidly. Faster erythrocyte processing of NO conceivably may reduce systemic NO (Figure 1D).

However, from the previous study, we did not find blood plasma levels of NO_2_^−^ and NO_3_^−^ to be significantly different between GP.Mur+ and GP.Mur-negative athletes (Hsu et al., 2021), although we also did not ask the study participants to have the same diet. Various factors, including individual diet preferences, could dynamically affect their blood NOx levels (NO_2_^−^ + NO_3_^−^). NO bioavailability can be increased in our body with consumption of more leafy green vegetables and beetroot (and even more nitrate-cured meats) in the diet.

On the other hand, fractional exhaled NO (FeNO) was affected by GP.Mur. FeNO was measured using NIOX, a clinical instrument that is intended to measure NO generated from Th2-driven inflammatory iNOS activities for the diagnosis of asthma (Menzies-Gow et al., 2020). We challenged the college athletes with an exhaustive running test protocol and measured their FeNO before the run and at the first and the fourth minute post-run. Their FeNO values decreased substantially from the exhaustive run and then rebounded gradually during the recovery phase, indicating a greater need for NO for vasodilation as intense exercise increased blood flow and gas exchange (Table 1: nearly 10-fold increases in minute ventilation [V_E_]). Both FeNO and the minute volume of exhaled NO (VNO = V_E_ * FeNO) were generally lower in GP.Mur+ than GP.Mur-negative athletes, and the differences reached statistical significance immediately after exercise (Table 1: 1-min post-run measurements). iNOS-associated type II airway inflammation was not involved, and clinical asthma was not found in these athletes since none of them showed >50 ppb FeNO. Thus, GP.Mur/increased AE1 affected exhaled NO (FeNO), which reflects lower NO in the pulmonary vasculature, especially during exercise-expanded pulmonary circulation (Table 1).

**TABLE 1:** FeNO from GP.Mur+ versus non-GP.Mur college athletes before and after an exhaustive running test. Minute ventilation (V_E_) was measured by cardiopulmonary exercise testing (CPET), which was immediately followed by FeNO measurement within a minute post-run. Statistical testing was performed by unpaired *t*-test. *n.s*., not significant. The study was approved by the MMH Institutional Review Board (MMH-IRB registration: 19MMHIS081e).

Group (N)	Non-GP.Mur (36)	GP.Mur (15)	*p*-value
Pre-run FeNO (ppb)	31.3 ± 22.4	21.3 ± 14.8	*n.s.*
1 min post-run FeNO (ppb)	25.5 ± 17.1	15.7 ± 10.7	*p < 0.05
4 min post-run FeNO (ppb)	26.5 ± 16.8	17.9 ± 11.8	*n.s.*
Pre-run V_E_ (L/min)	13.5 ± 3.1	16.9 ± 4.8	*p < 0.05*
0–1 min post-run V_E_ (L/min)	132.3 ± 24.3	145.5 ± 17.1	*P ∼ 0.06*
Pre-run VNO (nL/min)	339.0 ± 242.7	274.0 ± 171.0	*n.s.*
0–1 min post-run VNO (nL/min)	2771.3 ± 1983.1	1847.0 ± 1134.3	*p < 0.05

Although the levels of blood plasma NO metabolites were not different between GP.Mur and non-GP.Mur college athletes (Hsu et al., 2021), in our recently developed *GYP.Mur* knock-in (GPMur KI) B6J mice, significantly reduced NO bioavailability and early-onset hypertension were observed (unpublished data). Unlike the human study (Hsu et al., 2021), GPMur KI and the wildtype B6J mice are all bred under the same condition and fed with the same chow. The new murine data support the hypothesis that GP.Mur/more AE1 could contribute to reduced NO bioavailability. Perhaps by means of reduction of blood NO bioavailability (e.g., nitrate), the individual blood pressure setting could be elevated. The eNOS knock-out mice also show similar phenotypes—reduced blood NO bioavailability and hypertension (Wood et al., 2013).

## AE1 transports NO_2_^−^/NO_3_^−^ for erythrocyte processing of NO

To examine the effects of AE1-mediated NO_2_^−^/NO_3_^−^ transport on intraerythrocytic processing of NO species, we treated human whole blood samples (containing plasma and blood cells) with excessive nitrite (1 mM, compared to the generally submicromolar nitrite in human blood). The excessively added nitrite in the whole blood sample decreased from 1 mM to 10 μM within 20 min, indicating a rapid scavenging and metabolism of NO_2_^−^ by Hb. Using live-cell image recording with NO-sensitive fluorophore DAF, a surge of DAF fluorescence inside the RBCs appeared visibly within 1–2 min after adding excessive NO_2_^−^ in the milieu (Chen et al., 2024). If erythroid anion transport was first blocked by AE1-targeting antibodies or was delayed by replacing Cl^−^ (the counter-ion of AE1-mediated transport) with AE1-impermeant gluconate monoanion in the milieu, excessively added nitrite could remain imperishable in the blood plasma for a very long time. These demonstrate that inhibition of erythroid anion transport substantially reduces the rates of NO_2_^−^/NO scavenging and metabolism catalyzed by intraerythrocytic Hb (Chen et al., 2024).

## Discussion

From our human studies conducted in 2017–2022, we found that GP.Mur/increased AE1 is associated with early-onset hypertension. Non-diseased people with the GP.Mur blood type generally have slightly higher blood pressure, lower NO-dependent vasodilation, and lower fraction of exhaled nitric oxide (Table 1; Figure 1B, C) (Hsu et al., 2021; Chen et al., 2022). It has been a general impression among health workers in Eastern Taiwan that the local populations with higher percentages of GP.Mur are more susceptible to stroke and type II diabetes (T2D), though rigorous epidemiologic surveys have not been reported. Notably, hypertension is the major risk factor for both stroke and T2D.

To understand how the expression of glycophorin B-A-B variant GP.Mur on RBCs leads to higher blood pressure, we began to formulate a working model with the basic science that it is thermodynamically unfavorable for charged ions to permeate through the lipid bilayer (Figure 1A, D). More AE1 embedded in the RBC membrane makes the cells more permeable to monoanions and expedites NO_2_^−^/NO_3_^−^ membrane transport. NO_2_^−^ influx is particularly affected, as its concentration gradient across the red cell membrane is presumably much bigger due to deoxyHb-mediated NO_2_^−^ reduction to NO gas, which takes place inside the RBCs. When NO_2_^−^ enters an erythrocyte, NO_2_^−^ is also converted to N_2_O_3_, GSNO, and other NO species catalyzed by the Hb of different oxidation states (Patel et al., 2011). Thus, higher AE1 expression accelerates intraerythrocytic NO_2_^−^/NO metabolism, driving the conversion of NO_3_^−^ to NO_2_^−^ by microbiota nitrate reductases and xanthine oxidoreductase (Kapil et al., 2013); this conceivably also lowers plasma nitrate.

Since GP.Mur/increased AE1 is associated with blood NOx reduction, could blockade of AE1 instead help maintain NO bioavailability? Dipyridamole is an old anti-thrombotic and antianginal drug that blocks erythroid AE1 (Allahham et al., 2022). Dipyridamole reduces platelet aggregation and potentiates vasodilation with multiple mechanisms; its most well-studied mechanism for vasodilation is inhibition of adenosine receptors (Gamboa et al., 2005; Allahham et al., 2022). Whether dipyridamole as an AE1 inhibitor could also potentiate vasodilation has not been investigated because the link between AE1 and NO has been unclear for a long time. Dipyridamole, as a blocker of adenosine receptors, reduces the cellular uptake of adenosine and increases extracellular adenosine concentrations, which stimulates the activities of adenosine cyclase to generate more cAMP to potentiate eNOS activities and prostacyclin production (Gamboa et al., 2005; Allahham et al., 2022). Nonetheless, the transcript level of AE1 in human whole blood is 3-fold or higher than that of adenosine receptors (78.9 transcripts per million or TPM [AE1] versus 24.3 TPM [ADORA2A] from the human GTEx consortium) (Consortium et al., 2017). With our recent findings (Hsu et al., 2021; Chen et al., 2022; Chen et al., 2024), it is thus possible that dipyridamole-induced vasodilation could also be due to inhibition of erythroid AE1.

Dipyridamole binds to the AE1 dimer in a 1:1 stoichiometry and with a high affinity (K_d_ ∼1.2 μM) (Falke and Chan, 1986). As a secondary effect of AE1 inhibition, dipyridamole indirectly reduces K+ efflux from RBCs. Clinically, this function of dipyridamole protects sickle RBCs from excessive cation efflux and consequent dehydration (Joiner et al., 2001). Through the same mechanism, dipyridamole also protects RBC concentrates (i.e., as a transfusion product) from K+ leakage caused by virus-inactivating photosensitizer treatments (vanSteveninck et al., 2000). In the latter example, AE1–dipyridamole binding not only protects RBCs from K+ leak-associated hemolysis but also from peroxidative damage (Nepomuceno et al., 1997; van; Steveninck et al., 2000). Since AE1 is functionally versatile, without a doubt, our current model of AE1 in blood NO processing and vasodilation (Figure 1D) is far from complete.

Similar to the finding that GP.Mur increases erythroid HCO_3_^−^ permeability and respiratory excretion of CO_2_ (Hsu et al., 2009; Hsu et al., 2015; Hsu et al., 2023), here, we proposed that GP.Mur also increases erythroid NO_3_^−^/NO_2_^−^ permeability to accelerate erythrocyte processing of NO metabolites, which affects blood pressure (Patel et al., 2011) (Figure 1D). Could the two distinct anion transport modes of AE1 be coupled and AE1 serve as a coordinator for the seemingly separate physiological processes? A recently emerged clue is that GP.Mur+ carriers tend to rely more on aerobic respiration during intense exercise (Hsu et al., 2023). As intense physical activities demand more O_2_ delivery and blood flow, conceivably intravascular NO is metabolized and dissipated faster. With the new GPMur KI mouse model that recapitulates the hypertensive phenotype of GP.Mur+ people, we hope to uncover these physiologic interplays and health relevance stemming from the AE1-mediated transport of HCO_3_^−^/Cl^−^ and NO_3_^−^/NO_2_^−^ in the future.

## Data Availability

The raw data supporting the conclusion of this article will be made available by the authors, without undue reservation.
